# Membrane-initiated estrogen receptor-α signaling in the hypothalamus regulates trabecular bone in femur in female mice

**DOI:** 10.1530/JOE-25-0462

**Published:** 2026-05-20

**Authors:** Yiwen Jiang, Karin Horkeby, Petra Henning, Karin H Nilsson, Jianyao Wu, Lei Li, Sofia Movérare-Skrtic, Claes Ohlsson, Marie K Lagerquist

**Affiliations:** ^1^ Sahlgrenska Osteoporosis Centre, Centre for Bone and Arthritis Research at Institute of Medicine, Sahlgrenska Academy at University of Gothenburg, Gothenburg, Sweden; ^2^ Department of Drug Treatment, Sahlgrenska University Hospital, Region Västra Götaland, Gothenburg, Sweden

**Keywords:** estrogen receptor alpha, estrogenic effects, osteoporosis, bone tissue, central nervous system, neurons

## Abstract

Membrane-initiated estrogen receptor-α (mERα) signaling has been demonstrated to be crucial for normal bone metabolism, and our previous work has confirmed its essential role in osteoblasts. However, the contribution of brain-derived mERα signaling to bone homeostasis remains unexplored. To investigate the role of brain-derived mERα signaling in bone metabolism, we developed a POMC-C451A^f/f^ mouse model in which mERα signaling is selectively inactivated in POMC-expressing neurons. Gonadal-intact female POMC-C451A^f/f^ mice exhibited disturbed sex steroid levels and significantly increased bone mass in both cortical and trabecular compartments compared to littermate C451A^f/f^ controls after sexual maturation (16 weeks old). In ovariectomized female POMC-C451A^f/f^ mice, estradiol (E2) treatment enhanced the anabolic response in trabecular bone of the femur compared to controls, while the estrogen response in cortical bone was similar between the genotypes. Gonadal-intact male POMC-C451A^f/f^ mice displayed altered levels of testosterone compared to controls at 24 weeks of age. In orchiectomized male mice, responses to E2 treatment were similar across all examined parameters between POMC-C451A^f/f^ and control mice. In conclusion, our findings reveal an important role of membrane-initiated ERα signaling in POMC neurons in regulating hormone balance and bone metabolism, with more pronounced effects in female mice.

## Introduction

Estrogen plays a crucial role in bone metabolism by taking part in regulating the balance between bone formation and resorption (1). Estrogen exerts its bone-sparing effects through estrogen receptors, mainly estrogen receptor alpha (ERα) (2, 3), which is expressed in many tissues and cell types (4). The age-associated decline in circulating estrogen levels leads to disturbed bone metabolism in both men and women (5), particularly in women during menopause (6), causing enhanced bone resorption and increased risk of osteoporosis (7, 8, 9). Given the potential side effects associated with hormone replacement therapy, such as thromboembolism and reproductive cancers (10, 11), ongoing research is focused on developing safer and more effective tissue-specific estrogen therapies to treat osteoporosis.

In addition to the local effects mediated by ERα signaling in bone cells (7, 8, 9), bone mass can also be regulated by ERα signaling in the central nervous system (CNS) (12, 13, 14). Our previous study using the nestin-ERα^−/−^ mouse model to ablate ERα signaling in nervous tissue revealed a significant increase in both cortical and trabecular bone mass in female nestin-ERα^−/−^ mice, indicating that ERα signaling in the neuronal cells exerts an inhibitory effect on bone mass (12). Other studies using conditional knock-out models have also demonstrated that ERα signaling in various neuronal regions has different effects on bone mass (13, 14, 15). The hypothalamus, which is a part of the hypothalamic–pituitary–gonadal axis (HPGA) where ERα is highly expressed (16, 17), has been reported to be involved in the regulation of bone mass (18, 19, 20). Transgenic mouse models blocking ERα signaling in hypothalamic neurons have revealed an important role of ERα signaling in the hypothalamus for the regulation of bone mass and mechanical strength in female mice (13, 14). These mouse models targeted ERα signaling in POMC neurons or kisspeptin (Kiss1) neurons in the hypothalamus. POMC neurons are mostly located in the arcuate nucleus (ARC) region in the medial basal hypothalamus (MBH) (21), while Kiss1 neurons are located in both ARC and the ventromedial hypothalamus (VMH) regions (14). It was also reported that silencing ERα signaling in the VMH neurons alone does not influence bone mass (13), suggesting a more significant role of ERα signaling in the ARC in regulating bone mass. ERα is also expressed in brain regions outside the hypothalamus (17). A mouse model inhibiting ERα signaling in extrahypothalamic regions showed increased bone size and mechanical strength in female mice, but no effect on bone mass, with no similar effects observed in male mice (15). Collectively, these findings demonstrate that ERα signaling in hypothalamic neurons, especially in the ARC region, exerts a critical inhibitory effect on bone metabolism in female mice, with limited evidence of such an effect in male mice.

Membrane-initiated ERα signaling has gained increasing attention due to its regulatory effects on bone mass, as well as its tissue-specific and dose-dependent actions (22, 23, 24, 25, 26). Whereas nuclear ERα drives transcriptional regulation of estrogen-responsive genes, mERα activates rapid, non-genomic signaling cascades at the cell membrane, enabling distinct physiological responses (27). Notably, the skeletal response to E2 treatment is completely abolished in ERαAF-2^0^ mice lacking nuclear ERα activity, while it is dose-dependently reduced in mERα-deficient mice, highlighting that membrane estrogen receptor alpha (mERα) provides a unique and complementary role of estrogen signaling in skeletal regulation (3, 26).

Previously, we reported on a novel transgenic mouse model for conditional knockout, ERα-C451A^f/f^, designed to inactivate mERα signaling in targeted cell types (28). Using this model together with *Runx2*-Cre mice (29), we found that in female mice, the absence of mERα signaling in *Runx2*-expressing osteoblast lineage cells leads to reduced cortical bone mass and diminished mechanical strength by impacting osteoblast differentiation. Meanwhile, mERα signaling in hematopoietic cells is not involved in this regulatory process (28, 30), highlighting the cell-specificity of estrogen’s effects on bone physiology. It remains unclear whether mERα signaling in other cell types plays a role in the regulation of bone metabolism, particularly within neuronal populations such as POMC neurons, which are implicated in ERα-mediated central regulation of bone mass. Therefore, the aim of this study is to explore *in vivo* whether mERα signaling in the POMC neurons is involved in the regulation of bone metabolism.

## Methods

### Animals

All animals were housed in a standardized animal facility with a constant temperature of 22°C and a 12 h light:12 h darkness cycle. Mice were given a phytoestrogen-free pellet diet (Teklad diet 2016, Envigo, UK) and had access to tap water *ad libitum*. The animal experiments were approved by the Ethical Committee for Animal Research in Gothenburg and reported according to the ARRIVE guidelines.

To generate mice with specific inactivation of mERα signaling in the hypothalamic POMC neurons (POMC-C451A^f/f^), previously described ERα-C451A^f/f^ mice (denoted C451A^f/f^ mice) were crossed with POMC-Cre transgenic mice (21). In this study, homozygous C451A^f/f^ littermates were used as controls, as they have previously been shown to be phenotypically comparable to wild-type mice (28).

To rule out potential effects of Cre recombinase expression, we did a separate experiment comparing POMC-Cre mice with wild-type littermates. Body and tissue weights, sex steroid levels, and body composition were assessed in female and male POMC-Cre mice and their wildtype littermates at twelve weeks of age (Supplementary Tables 1 and 2 (see section on Supplementary materials given at the end of the article)). Primers used for genotyping are listed in Supplementary Table 3.

To validate the POMC-C451A^f/f^ model, DNA was isolated from the hypothalamus, liver, and cortical bone of eight-week-old POMC-C451A^f/f^ and littermate control female mice using DNeasy Blood & Tissue Kits (Qiagen, Germany). PCRs were performed with the listed primers (Supplementary Table 3), and the results were visualized on gel electrophoresis (Supplementary Fig. 1).

To observe the phenotypic differences between POMC-C451A^f/f^ and control mice, studies of gonadal-intact mice, without or before any interventions, were conducted. Gonadal-intact female mice were terminated and examined at 16 weeks of age. Body weight and body composition were also examined at five weeks of age in a separate experiment.

To assess the response to estrogen treatment, female POMC-C451A^f/f^ and homozygous C451A^f/f^ control mice were ovariectomized at 16 weeks of age. Male POMC-C451A^f/f^ mice and littermate controls were either sham-operated or orchiectomized at 20 weeks of age. All surgery procedures were performed under anesthesia with isoflurane (Baxter Medical AB, Sweden). Metacam (Boehringer Ingelheim Animal Health, Germany) was used as a postoperative analgesic. After one week of recovery, the mice received daily subcutaneous injections for three consecutive weeks with either vehicle (veh; Miglyol 812; OmyaPeralta GmbH, Germany), or low-dose 17β-estradiol-3-benzoate (E2, 1 μg/mouse/day, Sigma-Aldrich, USA), or high-dose E2 (6 μg/mouse/day, Sigma-Aldrich). Doses were chosen based on a previous study (28). Male sham-operated mice also received veh treatment. Mice were divided into body weight-matched groups prior to the administration of different treatments. In the female study, each treatment group contained 10–12 mice, while in the male study, each group consisted of 8–10 mice.

At termination, all mice were anesthetized using Ketador/Dexdomitor (Richter Pharma, Austria/Orion Pharma, Finland), bled from the axillary artery, and euthanized by cervical dislocation. Uterus, gonadal fat, and retroperitoneal fat were dissected and weighed. The femur and L5 vertebra were dissected, fixed in 4% paraformaldehyde for two days, and then stored in 70% ethanol for further phenotype analysis. Liver and hypothalamus tissue were dissected, snap-frozen in liquid nitrogen, and stored at −80°C for further analysis.

### Measurement of reproductive hormones

Serum concentrations of sex steroids were measured by high-sensitivity liquid chromatography–tandem mass spectrometry (LC–MS/MS) as previously described (31). The lower limits of quantification (LLOQ) for estradiol, testosterone, and progesterone are 0.5 pg/mL, 5 pg/mL, and 5 pg/mL, respectively, for undiluted samples. Concentrations below LLOQs were analyzed as half of the LLOQ. Serum concentration of the pituitary hormone follicle-stimulating hormone (FSH) was measured using MILLIPLEX^®^ Mouse Pituitary Panel (Merck Millipore, USA) according to the manufacturer’s protocol.

### Estrous cycle monitoring

The estrous cycle was monitored in four 24-week-old female POMC-C451A^f/f^ mice and four littermate controls by performing vaginal smears each morning for ten consecutive days. The smears were stained by Wright-Giemsa Stain (Sigma-Aldrich), and the determination of estrous stage was based on cytology as described (32).

### Measurement of bone turnover markers

As a bone formation marker, serum concentrations of procollagen type 1 N-terminal propeptide (P1NP) were measured using a Rat/Mouse EIA Kit (Immunodiagnostic Systems, UK). As a bone resorption marker, serum concentrations of C-terminal type 1 collagen (CTX-1) were measured using an ELISA RatLaps Kit (Immunodiagnostic Systems).

### RNA isolation and real-time PCR

Total RNA from the liver and hypothalamus was isolated using the RNeasy Mini Kit (Qiagen). The isolated RNA was reverse transcribed into cDNA using the high-capacity cDNA Reverse Transcription kit (Applied Biosystems, USA). Real-time PCR amplification was performed by Applied Biosystem StepOnePlus Real-Time PCR System (Thermo Fisher Scientific). The Assay-on-Demand probe sets (Thermo Fisher Scientific) used in this study included Estrogen receptor alpha (*Esr1*: Mm00433147_m), major urinary protein 3 (*Mup3*: Mm01702819_m1), prolactin receptor (*Prlr*: Mm0059957_m1), nephroblastoma overexpressed gene (*Nov*/*Ccn3*: Mm00456855_m1), and 18S (4310893E). The relative expression of target genes measured by probes was normalized to 18S and calculated using the 2^−ΔΔCt^ method. Primers used in this study included Efr3 homolog b (*Efr3b*:PrimerBank-MGH-PGA ID: 126723101c2), myosin VII a (*Myo7a*: PrimerBank-MGH-PGA ID: 367460065c1), and beta-actin (*Actb:* PrimerBank-MGH-PGA ID:6671509a1) with detailed sequences shown in Supplementary Table 3. These primers were measured using SYBR Green-based quantitative PCR. The relative expression of these genes was normalized to *Actb* and calculated using the 2^−ΔΔCt^ method.

### RNA sequencing and data analysis

Total RNA from the hypothalamus used for sequencing was isolated from 16-week-old female mice as described above. The quantity and quality of RNA isolated from the hypothalamus were determined using the 4200 TapeStation Automated Electrophoresis System (Agilent Technologies, USA). All samples had an RNA integrity number greater than 8. For each genotype, hypothalamic RNA from eight animals was randomly pooled in pairs, resulting in four samples per genotype. Sequencing libraries were constructed using the Illumina Stranded mRNA Library Preparation Kit with poly(A) mRNA enrichment and sequenced on an Illumina NovaSeq 6000 platform, generating paired-end 150 bp reads (2 × 150 bp) with the NovaSeq 6000 SP Reagent Kit (300 cycles, v1.5). Sequence quality was assessed by FastQC v0.12.1. RNA sequencing (RNA-seq) reads were quantified at the transcript level using Salmon (v1.10) with selective alignment against the GENCODE mouse reference transcriptome (vM32). Transcript-level abundance estimates were imported into R (v4.4) and summarized to gene-level counts using the tximport package (v1.28) based on transcript-to-gene annotations. Differential expression analysis between POMC-C451A^f/f^ and control samples was performed using DESeq2 (v1.40). Multiple testing correction was applied using the Benjamini–Hochberg method, and genes with a false discovery rate (FDR) ≤ 0.05 were considered differentially expressed. A variance-stabilizing transformation (VST) was applied to normalized counts for principal component analysis and heatmap visualization. Heatmaps were generated using the pheatmap package (v1.0.12), with genes clustered based on correlation distance and samples annotated by genotype. RNA-seq data, including raw data files (fastq format) and processed data files (matrix table of gene counts and differentially expressed genes in CSV format), are available in the Gene Expression Omnibus (GEO) repository, accession number GSE328990.

### Assessment of bone parameters

#### Dual-energy X-ray absorptiometry

Total body areal bone mineral density (aBMD), lean mass, and fat percentage were analyzed using a Lunar PIXImus mouse densitometer (Wipro GE Healthcare, USA).

#### Peripheral quantitative computed tomography

Peripheral quantitative computed tomography (pQCT) was performed on the femurs using the pQCT XCT RESEARCH M (version 4.5B, Norland) at a resolution of 70 μm as previously described (33). Trabecular bone was analyzed by a scan positioned in the metaphysis at a distance from the distal growth plate corresponding to 3% of the total length of the femur. The trabecular bone region was defined as the inner 45% of the total cross-sectional area. Cortical bone was scanned and analyzed in the mid-diaphyseal region at a distance from the distal growth plate corresponding to 36% of the total length of the femur.

#### High‐resolution microcomputed tomography

High-resolution microcomputed tomography (μCT) analysis was performed on femurs and L5 vertebrae using the SkyScan 1275 model (Bruker MicroCT, Belgium). The scanning and analysis settings were conducted as previously described (28). Briefly, in the vertebrae, trabecular and cortical analyses were initiated 7 μm caudal to the pedicles, extending 245 μm longitudinally, with regions of interest manually defined. In the femurs, cortical analysis was conducted 5.2 mm from the distal growth plate, extending 210 μm proximally. The trabecular analysis in the femur, excluding cortical bone, started 504 μm from the distal growth plate, extending 210 μm proximally.

#### Dynamic bone histomorphometry

Dynamic histomorphometry was performed using calcein and alizarin labeling. Mice received intraperitoneal injections of calcein and alizarin (Merck GmbH, Germany) nine and two days prior to termination, respectively. Following dissection, left femurs were fixed in 4% formaldehyde for 48 h, stored in 70% EtOH until dehydrated through a graded ethanol series, incubated overnight in 30% sucrose, and embedded in OCT compound. Longitudinal 10 μm thick cryosections spanning the entire femur were prepared using Kawamoto’s film method (34) with Cryofilm type 3C (16UF). Fluorescent images were acquired using a Nikon spinning-disk microscope. Dynamic histomorphometric analyses were performed using ImageJ according to the guidelines of the American Society for Bone and Mineral Research (ASBMR) (35). The region of interest was defined in the secondary spongiosa of the distal femur, beginning 200 μm below the growth plate and extending distally to the midpoint of the femur. Within the region of interest, trabecular bone was analyzed for standard dynamic bone formation parameters, including mineral apposition rate (MAR), mineralizing surface per bone surface (MS/BS), and bone formation rate per bone surface (BFR/BS).

### Statistical analysis

In the gonadal-intact studies, differences between female POMC-C451A^f/f^ and control mice were assessed using Student’s *t*-test (Excel). When the *F*-test indicated significantly unequal variances between groups, Welch’s *t*-test was applied (GraphPad Prism, USA, version 10.2.2). In the treatment studies, the effects of genotype and different treatments, as well as their interaction, were assessed using two-way ANOVA. This was followed by Tukey’s multiple comparison test to examine the differences between POMC-C451A^f/f^ and control mice receiving the same treatment and the differences between vehicle and E2-treated groups in the same genotype (GraphPad Prism). In all analyses, a difference was considered significant when *P* < 0.05. Data normality was assessed using the Shapiro–Wilk test (GraphPad Prism). Non-normally distributed data were log-transformed to achieve normality and reduce heteroscedasticity. Outliers were identified and excluded using Grubbs’ test (GraphPad Prism). In some analyses, the number of samples was fewer than the number of animals because certain samples were lost or insufficient for measurement. Values in the figures (visualized using GraphPad Prism) are displayed as mean ± SEM, while the tables present values as mean ± SD.

## Results

### Gonadal-intact female POMC-C451A^f/f^ mice manifest disturbed sex steroid levels and increased bone mass

To confirm the specificity of the animal model, tissue-specific recombination of the ERα-C451A mutation was validated by PCR analysis. The mutation was detected in DNA from the hypothalamus but was absent in DNA from liver and cortical bone tissues of POMC-C451A^f/f^ mice, with tissues from C451A^f/f^ littermates serving as negative controls (Supplementary Fig. 1A). mRNA expression of total *Esr1* in the hypothalamus was unaffected by the mutation in POMC-C451A^f/f^ mice (Supplementary Fig. 1B).

**Figure 1:**
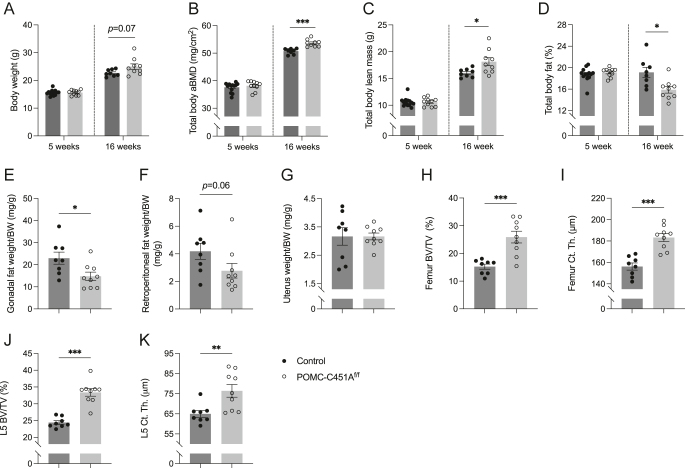
Increased bone mass and reduced adipose tissue in gonadal-intact female POMC-C451A^f/f^ mice. Female POMC-C451A^f/f^ and control mice were weighed (A), and body composition was measured by dual-energy X-ray absorptiometry at 5 and 16 weeks of age (two separate experiments), including total body areal bone mineral density; aBMD (B), total body lean mass (C), and total body fat percentage (D). At 16 weeks of age, gonadal fat (E), retroperitoneal fat (F), and uterus (G) were dissected and weighed. The femur and L5 vertebra were also collected and analyzed by high-resolution microcomputed tomography to assess femur trabecular bone volume fraction (BV/TV) (H), femur cortical thickness (Ct. Th.) (I), L5 BV/TV (J), and L5 Ct. Th. (K). The difference between femur BV/TV of POMC-C451A^f/f^ and control mice was analyzed by Welch’s *t*-test. Others were analyzed by Student’s *t*-test. Data are presented as mean ± SEM with individual values as dots. *P < 0.05, **P < 0.01, ***P < 0.001. BW, body weight. Five-week-old control *n* = 13, 5-week-old POMC-C451A^f/f^ *n* = 10, 16-week-old control *n* = 8, and 16-week-old POMC-C451A^f/f^ *n* = 9.

At five weeks of age (prepubertal), female POMC-C451A^f/f^ and control mice exhibited similar body weights and body composition parameters, including total body aBMD, lean mass, and fat percentage (Fig. 1A, B, C, D). After sexual maturation, at 16 weeks of age, female POMC-C451A^f/f^ mice showed a trend toward increased body weight, and significant alterations were detected in body composition, including increased total body aBMD, increased lean mass, and reduced fat percentage compared to control mice (Fig. 1A, B, C, D). Consistent with the reduction in fat percentage, gonadal fat weight normalized to body weight (BW) was significantly decreased in female POMC-C451A^f/f^ mice compared to controls, with a similar trend toward decreased retroperitoneal fat weight/BW (Fig. 1E and F). No difference was observed in uterus weight/BW between the genotypes (Fig. 1G).

Further bone analyses at 16 weeks of age revealed significant increases in both trabecular and cortical bone in the femur in female POMC-C451A^f/f^ mice compared to controls (Fig. 1H and I, Table 1). Trabecular changes were characterized by increased trabecular bone volume fraction (BV/TV), trabecular thickness, and trabecular number, and reduced trabecular separation (Fig. 1H, Table 1). Cortical changes were characterized by increased cortical thickness (Fig. 1I) and cortical area, reduced endosteal circumference, while periosteal circumference remained unchanged (Table 1). Significant effects on both trabecular and cortical bone were also observed in the L5 vertebra of female POMC-C451A^f/f^ mice compared to controls (Fig. 1J and K). However, serum levels of the bone turnover markers P1NP and CTX-1 did not differ between gonadal-intact female POMC-C451A^f/f^ mice and controls (Table 1).

**Table 1:** Trabecular and cortical bone parameters in the femur and serum levels of bone turnover markers from gonadal-intact female mice.

	Control	POMC-C451A^f/f^
Tb.Th. (μm)	61.3 ± 4.9	68.3 ± 6.2*
Tb.Sp. (μm)	185 ± 9	163 ± 13***
Tb.N. (1/mm)	2.48 ± 0.36	3.75 ± 0.71***
Ct.Ar. (mm^2^)	0.63 ± 0.03	0.73 ± 0.05***
Endo-C (mm)	3.51 ± 0.09	3.39 ± 0.12*
Peri-C (mm)	4.49 ± 0.07	4.54 ± 0.11
P1NP (ng/mL)	49.3 ± 10.0	44.6 ± 15.3
CTX-1 (ng/mL)	18.1 ± 5.1	16.1 ± 7.6

Femurs from gonadal-intact female POMC-C451A^f/f^ (*n* = 9) and control (*n* = 8) were assessed by micro-computed tomography (μCT) at 16 weeks of age. Serum levels of bone turnover markers were measured by ELISA in POMC-C451A^f/f^ (*n* = 8) and controls (*n* = 8). The differences between POMC-C451A^f/f^ and control mice were analyzed by Student’s *t-*test. All values are presented as mean ± SD. *P < 0.05, ***P < 0.001. Tb.Th., Trabecular thickness; Tb.Sp., trabecular separation; Tb.N., trabecular number; Ct.Ar., cortical area; Endo-C, endosteal circumference; Peri-C, periosteal circumference; P1NP, procollagen type 1 N-terminal propeptide; CTX-1, C-terminal type 1 collagen.

Growth hormone signaling is an important regulator of bone growth and bone mass (36), which is also known to be affected by estrogen receptor signaling (37). To investigate whether loss of mERα signaling in POMC neurons affects the growth hormone secretion pattern, we measured mRNA expression of major urinary protein 3 (*Mup3*) and prolactin receptor (*Prlr*) in the liver. However, no significant changes in expression were observed compared to controls (Supplementary Fig. 2A and B). Furthermore, no significant difference in hypothalamic mRNA expression of the bone-related nephroblastoma overexpressed gene (*Ccn3*) (38) was detected in female POMC-C451A^f/f^ mice compared to controls (Supplementary Fig. 2C). RNA-seq analysis of RNA from the hypothalamus identified 18 differentially expressed genes (DEGs) between female POMC-C451A^f/f^ and control mice, defined by |log_2_(fold change)| > 0.5 and a FDR ≤ 0.05 (Supplementary Table 4; Supplementary Fig. 3A). Among these DEGs, *Efr3b* and *Myo7a* have previously been implicated in the regulation of bone mass according to data from the International Mouse Phenotyping Consortium (IMPC) (39, 40). qPCR validation confirmed the RNA-seq findings, demonstrating upregulation of *Efr3b* and downregulation of *Myo7a* in the hypothalamus of female POMC-C451A^f/f^ mice compared to controls (Supplementary Fig. 3B and C).

At 16 weeks of age, female POMC-C451A^f/f^ mice manifested moderately elevated serum levels of estradiol and testosterone, while progesterone levels increased more than tenfold compared to the controls (Table 2). In contrast, the pituitary hormone FSH remained unchanged (Table 2), consistent with the finding in POMC-ERαKO mice (21). Monitoring of the estrous cycle at six months of age revealed a disturbed cycle with shortened estrous phase and prolonged diestrus phase in female POMC-C451A^f/f^ mice compared to controls (Supplementary Fig. 4).

**Table 2:** Circulating levels of hormones in gonadal-intact female mice.

	Control	POMC-C451A^f/f^
Estradiol (pg/mL)	3.08 ± 2.56	8.44 ± 4.42**
Testosterone (pg/mL)	12.36 ± 7.31	32.33 ± 11.72***
Progesterone (pg/mL)	1,096 ± 1,151	13,313 ± 8,697**
FSH (pg/mL)	1,036 ± 980	471 ± 236

Serum concentrations of estradiol, testosterone, and progesterone of gonadal-intact female POMC-C451A^f/f^ (*n* = 9) and control (*n* = 8) were analyzed by high-sensitivity liquid chromatography–tandem mass spectrometry at the age of 16 weeks. Serum concentration of follicle-stimulating hormone (FSH) was also measured in POMC-C451A^f/f^ (*n* = 8) and controls (*n* = 8). The differences in serum estradiol and testosterone between POMC-C451A^f/f^ and control mice were analyzed by Student’s *t-*test. The differences in serum progesterone and FSH were analyzed by Welch’s *t-*test. All values are presented as mean ± SD. **P < 0.01, ***P < 0.001.

### Female POMC-C451A^f/f^ mice show an enhanced response to estradiol treatment in trabecular bone in femur

**Figure 2:**
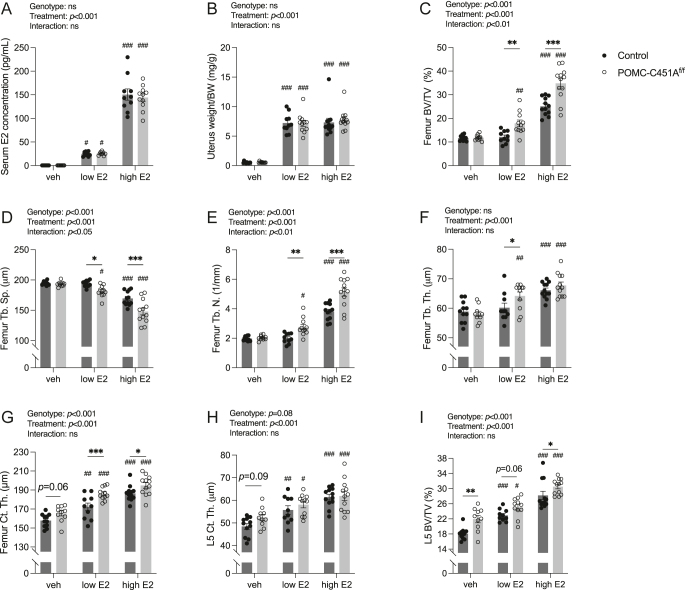
Enhanced estrogen response in trabecular bone in the femur of female POMC-C451A^f/f^ mice. Vehicle, low-dose estradiol (E2), and high-dose E2 were administered subcutaneously in ovariectomized POMC-C451A^f/f^ and control female mice. After three weeks of administration, serum E2 concentration (A) and uterus weight (B) were measured. Femur trabecular bone volume fraction (BV/TV) (C), trabecular separation (Tb.Sp.) (D), trabecular number (Tb.N.) (E), trabecular thickness (Tb.Th.) (F), femur cortical thickness (Ct.Th.) (G), lumbar vertebra 5 (L5) Ct.Th. (H), and L5 BV/TV (I) were assessed by high-resolution microcomputed tomography (μCT). Two-way ANOVA test was applied (results presented above each plot) followed by Tukey’s multiple comparison test to compare the differences between genotypes within each treatment group, and between vehicle- and E2-treated mice within the same genotype. A significant difference by Tukey’s multiple comparison test is indicated by *P < 0.05, **P < 0.01, ***P < 0.001 (between genotypes within the same treatment) and ^#^*P* < 0.05, ^##^*P* < 0.01, ^###^*P* < 0.001 (between vehicle- and E2-treated mice within the same genotype). Data are presented as mean ± SEM with individual values as dots. Vehicle control *n* = 11, vehicle POMC-C451A^f/f^ *n* = 10, low-dose E2 control *n* = 10, low-dose E2 POMC-C451A^f/f^ *n* = 11, high-dose E2 control *n* = 12, and high-dose E2 POMC-C451A^f/f^ *n* = 12.

Subcutaneous injections of vehicle, low-dose, or high-dose of estradiol (E2) were administered to ovariectomized female POMC-C451A^f/f^ and control mice for three weeks. Within each treatment group, POMC-C451A^f/f^ and control mice showed similar serum E2 concentrations, body weights, and uterus weight/BW (Fig. 2A and B, Supplementary Fig. 5A).

Both low- and high-dose E2 treatments significantly increased uterus weight/BW in POMC-C451A^f/f^ and control mice, with no differences in treatment responses observed between the genotypes (Fig. 2B). DXA analysis revealed similar significant changes in total body aBMD and fat percentage after low- and high-dose E2 treatment in both genotypes compared to vehicle treatment, while lean mass remained unaffected by E2 treatment (Supplementary Fig. 5B, C, D). An overall increase in total body aBMD and a decrease in fat percentage, indicated by significant genotype effects by ANOVA test, were detected in female POMC-C451A^f/f^ mice compared to controls, without a significant interaction between genotype and treatment (Supplementary Fig. 5B and D). Consistent with the observed differences in total fat percentage, E2 treatments also reduced gonadal fat weight/BW and retroperitoneal fat weight/BW in both genotypes (Supplementary Fig. 5E and F). Additionally, overall decreases in these dissected fat depot weights/BW were observed in female POMC-C451A^f/f^ mice compared to controls (Supplementary Fig. 5E and F).

**Figure 3:**
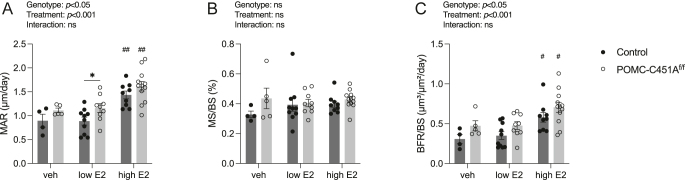
Elevated bone formation rate in female POMC-C451A^f/f^ mice. Vehicle, low-dose estradiol (E2), and high-dose E2 were administered subcutaneously in ovariectomized POMC-C451A^f/f^ and control female mice. They were sacrificed after three weeks of daily treatment at the age of 20 weeks. Dynamic bone histomorphometry analyses were performed in left femurs, including mineral apposition rate (MAR) (A), mineralizing surface per bone surface (MS/BS) (B), and bone formation rate per bone surface (BFR/BS) (C). Two-way ANOVA test was applied (results presented above each plot) followed by Tukey’s multiple comparison test to compare the differences between genotypes within each treatment group and between vehicle- and E2-treated mice within the same genotype. A significant difference by Tukey’s multiple comparison test is indicated by *P < 0.05 (between genotypes within the same treatment) and ^#^*P* < 0.05, ^##^*P* < 0.01 (between vehicle- and E2-treated mice within the same genotype). Data are presented as mean ± SEM with individual values as dots. Vehicle control *n* = 4, vehicle POMC-C451A^f/f^ *n* = 5, low-dose E2 control *n* = 10, low-dose E2 POMC-C451A^f/f^ *n* = 9, high-dose E2 control *n* = 9, and high-dose E2 POMC-C451A^f/f^ *n* = 12.

Notably, trabecular bone parameters in the femur in control mice (including BV/TV, separation, number, and thickness) did not respond to low-dose E2 treatment, whereas POMC-C451A^f/f^ mice showed significant changes in all the evaluated trabecular parameters (Fig. 2C, D, E, F). High-dose E2 treatment significantly affected all examined trabecular parameters in the femur in both genotypes (Fig. 2C, D, E, F). Importantly, POMC-C451A^f/f^ female mice demonstrated a significantly enhanced response to E2 treatment in trabecular BV/TV, separation, and number in the femur compared to the controls (Fig. 2C, D, E). Further bone analyses demonstrated that both low- and high-dose E2 treatments significantly increased cortical thickness and area in the femur and cortical thickness of L5 vertebrae in both genotypes, with comparable E2 responses between POMC-C451A^f/f^ and control mice (Fig. 2G and H, Table 3). Furthermore, the serum bone turnover markers P1NP and CTX-1 and dynamic histomorphometric analyses showed no significant differences in E2 responses between genotypes (Table 3, Fig. 3). However, there was a significant overall increase in trabecular bone formation in female POMC-C451A^f/f^ mice compared to controls, as shown by elevated MAR and BFR/BS (Fig. 3A and C). A significant overall increase of cortical thickness and area in the femur was detected in female POMC-C451A^f/f^ mice compared to controls, along with a similar trend toward an overall increase in cortical thickness of the L5 vertebra (Fig. 2G and H, Table 3). Endosteal circumference of femur in both genotypes responded to high-dose E2 treatment but not to low-dose E2, while periosteal circumference remained unchanged with either E2 treatment in both genotypes (Table 3). Analysis of the trabecular bone in L5 vertebra revealed similar results as for the cortical bone, with increased BV/TV after both low- and high-dose E2 treatments, as well as an overall increase in BV/TV when comparing POMC-C451A^f/f^ mice and controls regardless of treatments (Fig. 2I).

**Table 3:** Estrogen treatment responses in the femur and bone turnover markers in female mice.

	Vehicle		Low-dose E2		High-dose E2		Two-way ANOVA		
	Control	POMC-C451A^f/f^	Control	POMC-C451A^f/f^	Control	POMC-C451A^f/f^	Genotype	Treatment	Interaction
Ct.Ar. (mm^2^)	0.63 ± 0.02	0.67 ± 0.03*	0.68 ± 0.05^##^	0.75 ± 0.04***^,###^	0.74 ± 0.04^###^	0.77 ± 0.04*^,###^	*P* < 0.001	*P* < 0.001	ns
Endo-C (mm)	3.50 ± 0.12	3.51 ± 0.08	3.44 ± 0.09	3.45 ± 0.14	3.36 ± 0.12^#^	3.35 ± 0.14^##^	ns	*P* < 0.001	ns
Peri-C (mm)	4.49 ± 0.10	4.55 ± 0.08	4.52 ± 0.08	4.62 ± 0.14*	4.53 ± 0.11	4.58 ± 0.13	*P* < 0.05	ns	ns
P1NP (ng/mL)	41.8 ± 9.9	39.3 ± 8.3	46.0 ± 12.5	43.0 ± 14.8	58.0 ± 7.0^##^	59.6 ± 12.8^###^	ns	*P* < 0.001	ns
CTX-1 (ng/mL)	19.0 ± 3.3	17.7 ± 4.0	14.5 ± 3.1^#^	14.6 ± 3.9	19.1 ± 2.9	20.5 ± 3.9	ns	*P* < 0.001	ns

Ovariectomized female POMC-C451A^f/f^ and control mice received vehicle, low-dose estradiol (E2), or high-dose E2 treatment for three weeks. Cortical parameters of the femur were assessed by high-resolution microcomputed tomography (μCT). Serum levels of bone turnover markers were measured by ELISA. Two-way ANOVA test was applied, followed by Tukey’s multiple comparison test to compare differences between genotypes within each treatment group, and between vehicle- and E2-treated mice within the same genotype. A significant difference by Tukey’s multiple comparison test is indicated by *P < 0.05, ***P < 0.001 (between genotypes within the same treatment group) and ^#^*P* < 0.05, ^##^*P* < 0.01, ^###^*P* < 0.001 (between vehicle- and E2-treated mice within the same genotype). All values are presented as mean ± SD. Cortical area, Ct.Ar.; endosteal circumference, Endo-C; periosteal circumference, Peri-C; procollagen type 1 N-terminal propeptide, P1NP;C-terminal type 1 collagen, CTX-1. Vehicle control *n* = 11, vehicle POMC-C451A^f/f^ *n* = 10, low-dose E2 control *n* = 10, low-dose E2 POMC-C451A^f/f^ *n* = 11, high-dose E2 control *n* = 12, and high-dose E2 POMC-C451A^f/f^ *n* = 12.

### Male POMC-C451A^f/f^ and control mice exhibit similar responses to estrogen treatment

**Figure 4:**
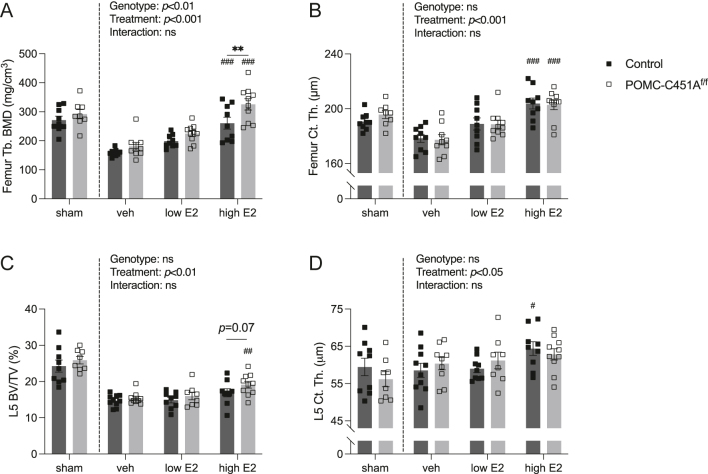
Male POMC-C451A^f/f^ and control mice exhibit similar bone responses to estrogen treatment. Vehicle, low-dose estradiol (E2), and high-dose E2 were administered subcutaneously in orchiectomized POMC-C451A^f/f^ and control male mice for three weeks. Sham-operated mice also received vehicle treatment for three weeks. At 24 weeks of age, all mice were sacrificed. Femur trabecular bone mineral density (Tb.BMD) (A) and femur cortical thickness (Ct.Th.) (B) were analyzed using peripheral quantitative computed tomography (pQCT). Lumbar vertebra 5 (L5) trabecular bone volume fraction (BV/TV) (C) and L5 Ct.Th. (D) were assessed by high-resolution microcomputed tomography (μCT). Student’s *t*-test was performed to verify the absence of significant differences between sham-operated POMC-C451A^f/f^ and control male mice. The differences between different treatments were analyzed by a two-way ANOVA test followed by Tukey’s multiple comparison test to compare the differences between genotypes within each treatment group and between vehicle- and E2-treated mice within the same genotype. A significant difference by Tukey’s multiple comparison test is indicated by **P < 0.01 (between genotypes within the same treatment) and ^#^*P* < 0.05, ^##^*P* < 0.01, ^###^*P* < 0.001 (between vehicle- and E2-treated mice within the same genotype). Data are presented as mean ± SEM with individual values as dots. Vehicle-treated sham control *n* = 9, vehicle-treated sham POMC-C451A^f/f^ *n* = 8, vehicle control *n* = 10, vehicle POMC-C451A^f/f^ *n* = 9, low-dose E2 control *n* = 9, low-dose E2 POMC-C451A^f/f^ *n* = 10, high-dose E2 control *n* = 9, and high-dose E2 POMC-C451A^f/f^ *n* = 10.

Sham-operated male POMC-C451A^f/f^ mice exhibited elevated serum testosterone levels compared to control mice at 24 weeks of age (Table 4). No other significant changes were observed between sham-operated male POMC-C451A^f/f^ mice and controls in serum sex steroid levels, body weights, body weight-adjusted weights of fat tissues, or trabecular and cortical bone parameters of the femur and L5 vertebrae (Fig. 4A, B, C, D, Table 4).

**Table 4:** Phenotypes of male POMC-C451A^f/f^ and control mice.

	Sham		Vehicle		Low-dose E2		High-dose E2		Two-way ANOVA		
	Control	POMC-C451A^f/f^	Control	POMC-C451A^f/f^	Control	POMC-C451A^f/f^	Control	POMC-C451A^f/f^	Genotype	Treatment	Interaction
Body weight (g)	33.8 ± 3.0	34.2 ± 1.4	28.6 ± 3.0	27.4 ± 2.5	28.6 ± 2.4	29.4 ± 1.8	28.5 ± 2.0	29.4 ± 1.6	ns	ns	ns
Gonadal fat weight/BW (mg/g)	34.3 ± 10.8	34.7 ± 14.0	33.4 ± 13.5	29.9 ± 11.8	21.4 ± 6.7^#^	24.2 ± 10.8	19.7 ± 7.3^#^	17.7 ± 10.2^#^	ns	*P* < 0.01	ns
Retroperitoneal fat weight/BW (mg/g)	11.6 ± 3.1	10.8 ± 2.8	10.1 ± 4.8	9.1 ± 3.4	6.9 ± 2.1	7.1 ± 3.3	5.2 ± 2.1^##^	4.4 ± 2.7^##^	ns	*P* < 0.001	ns
Testosterone (pg/mL)	2,448.1 ± 3,789.5	5,786.3 ± 9,050.1*	5.9 ± 1.6	5.3 ± 1.8	3.9 ± 1.7^#^	2.8 ± 0.9^##^	3.6 ± 1.7^##^	<LLOQ^###^	*P* < 0.05	*P* < 0.001	ns
Estradiol (pg/mL)	<LLOQ	<LLOQ	<LLOQ	<LLOQ	26.2 ± 8.2^###^	22.0 ± 4.6^###^	137.2 ± 17.4^###^	132.1 ± 18.8^###^	ns	*P* < 0.001	ns
Progesterone (pg/mL)	728 ± 553	552 ± 336	1,014 ± 649	972 ± 539	236 ± 198^###^	197 ± 152^###^	99 ± 68^###^	125 ± 74^###^	ns	*P* < 0.001	ns

Vehicle, low-dose estradiol (E2), and high-dose E2 were administered subcutaneously in orchiectomized POMC-C451A^f/f^ and control male mice for three weeks. Sham-operated mice also received vehicle treatment for three weeks. At 24 weeks of age, all mice were sacrificed. Mice were weighed, and gonadal fat tissue and retroperitoneal fat tissue were collected, weighed, and normalized to body weight (BW). Serum concentrations of testosterone, estradiol, and progesterone were measured using high-sensitivity liquid chromatography–tandem mass spectrometry. Differences between sham-operated POMC-C451A^f/f^ and control mice were analyzed by Student’s *t-*test. A significant difference by Student’s *t-*test is indicated by *P < 0.05. Differences between treatment groups were analyzed by two-way ANOVA test followed by Tukey’s multiple comparison test to compare the differences between genotypes within each treatment group and between vehicle- and E2-treated mice within the same genotype. A significant difference by Tukey’s multiple comparison test is indicated by ^#^*P* < 0.05, ^##^*P* < 0.01, ^###^*P* < 0.001 (between vehicle- and E2-treated mice within the same genotype). All values are presented as mean ± SD. Body weight, BW; non-significant, ns. Vehicle-treated sham control *n* = 9, vehicle-treated sham POMC-C451A^f/f^ *n* = 8, vehicle control *n* = 10, vehicle POMC-C451A^f/f^ *n* = 9, low-dose E2 control *n* = 9, low-dose E2 POMC-C451A^f/f^ *n* = 10, high-dose E2 control *n* = 9, and high-dose E2 POMC-C451A^f/f^ *n* = 10.

Both low- and high-dose E2 treatments induced significant changes in sex steroid levels compared to vehicle treatment in both genotypes (Table 4). An overall decrease in testosterone was observed in vehicle- and E2-treated orchiectomized male POMC-C451A^f/f^ mice compared to controls (Table 4). Skeletal changes were detected only with high-dose E2 treatment in both controls and POMC-C451A^f/f^ mice (Fig. 4). In both genotypes, high-dose E2 treatment increased trabecular BMD and cortical thickness in the femur (Fig. 4A and B). Interestingly, a significantly higher trabecular BMD in the femur was detected in high-dose E2-treated POMC-C451A^f/f^ male mice compared to controls (Fig. 4A). A trend toward higher BV/TV in L5 vertebra after high-dose E2 treatment was observed in POMC-C451A^f/f^ male mice compared to controls (Fig. 4C). However, the responses to E2 treatment did not differ significantly between POMC-C451A^f/f^ male mice and controls for any of the examined parameters (Fig. 4, Table 4).

## Discussion

Central ERα signaling has been identified as a key regulator of bone metabolism, particularly within the hypothalamus (12, 13, 14, 41, 42). In this study, we demonstrate that the loss of membrane-initiated ERα signaling in POMC-expressing neurons leads to altered sex steroid levels and increased bone mass in female POMC-C451A^f/f^ mice, along with enhanced sensitivity to E2 treatment in trabecular bone in the femur. Male mutants exhibited minimal changes, except for an increased trabecular bone mass in the femur after high-dose E2 treatment, compared to high-dose E2-treated male controls. These results highlight the role of mERα signaling in POMC neurons in regulating hormone balance and bone homeostasis.

Previous studies have shown that loss of ERα signaling in neuronal cells, either broadly as in nestin-ERαKO mice or within specific neuronal subsets, as in POMC-ERαKO and Nkx2-1-ERαKO mice, results in significantly increased cortical and trabecular bone mass in gonadal-intact female mice (12, 13, 14, 21). Interestingly, gonadal-intact female POMC-C451A^f/f^ mice also exhibit significantly increased bone mass in both cortical and trabecular compartments of the femur and L5 vertebra compared to controls. Thus, these findings indicate that mERα signaling in POMC neurons is a key factor for the regulation of the skeleton. The increase in cortical bone mass in female mice, characterized by decreased endosteal circumference and unchanged periosteal circumference (3), may be attributed to elevated serum E2 concentrations. However, considering that female POMC-C451A^f/f^ mice showed only a modest elevation in E2 serum levels compared to controls, remaining within the physiological range, comparable to global mERαKO (26) and lower than levels observed with total ERα deletion (43), it is possible that additional mechanisms also contribute to the observed bone phenotype. Previous studies indicate that POMC neurons are involved in regulating the expression of growth hormone, a known regulator of bone growth and bone mass (36, 37), as well as the secretion of brain-derived osteoanabolic hormone CCN3 (38). Nonetheless, no significant differences were observed in the mRNA expression in the liver of markers for growth hormone secretion patterns (44, 45), *Mup* and *Prlr*, or in hypothalamic mRNA expression of *Ccn3* between POMC-C451A^f/f^ mice and controls, indicating that these factors are unlikely to mediate the increased bone mass. Bulk RNA-seq analysis of the hypothalamus identified a total of 18 DEGs. Further annotation using the IMPC database indicated that two of these genes, *Efr3b* and *Myo7a*, both expressed in the hypothalamus (39, 40, 46, 47, 48), are associated with an altered skeletal phenotype. These findings suggest that the elevated bone mass observed in female POMC-C451A^f/f^ mice may be related to altered expression of *Efr3b* and *Myo7a* within the hypothalamus. However, because POMC neurons constitute a small fraction of all hypothalamic cells (49, 50), bulk RNA-seq may obscure cell type-specific gene expression changes occurring within this relatively small neuronal population. Future studies employing higher-resolution approaches will be valuable for uncovering additional molecular alterations within POMC neurons and for clarifying the mechanisms through which mERα signaling influences skeletal regulation.

To further investigate the role of mERα signaling in POMC neurons in regulating bone metabolism, we performed ovariectomy followed by vehicle, low- or high-dose E2 treatments to eliminate the confounding effects of endogenous estrogen in gonadal-intact mice and to assess responses to E2 in various tissues. In a previous study on ERα signaling in POMC neurons and its effects on E2 responses in bone, it was shown that loss of ERα signaling in POMC neurons in female mice resulted in enhanced estrogenic response in cortical bone in both femur and L5 vertebra, as well as a moderately augmented E2 response in trabecular bone in femur (13). In this study, we found that loss of mERα signaling in POMC neurons did not alter the E2 response in cortical bone in the femur. This suggests that the mediating effects of ERα signaling in POMC neurons on cortical bone metabolism are primarily driven by nuclear ERα signaling rather than membrane-initiated mechanisms. In contrast, the enhanced E2 response in trabecular bone in the femur of POMC-C451A^f/f^ females demonstrates that mERα signaling in POMC neurons has a clear inhibitory effect on this bone compartment. Furthermore, although the serum bone turnover markers P1NP and CTX-1 did not show significant differences in E2 responses, these circulating markers represent only a momentary snapshot of systemic bone turnover and may, therefore, fail to capture localized or compartment-specific alterations in bone formation. Interestingly, dynamic histomorphometric analyses revealed a significant overall increase in trabecular bone formation in female POMC-C451A^f/f^ mice, including elevated MAR and BFR/BS, demonstrating that the absence of mERα signaling in POMC neurons enhances the capacity of osteoblasts to form new bone. Together, these findings suggest that mERα signaling in POMC neurons functions as a negative regulator of trabecular bone formation by influencing osteoblast activity.

An overall increase in aBMD and a decrease in fat parameters were observed in POMC-C451A^f/f^ mice compared to controls, as shown by significant genotype effects from two-way ANOVA tests. At the time of termination, four weeks had passed since ovariectomy, by which time residual endogenous estrogen levels were known to be significantly diminished (43). However, since differences were present between vehicle-treated ovariectomized POMC-C451A^f/f^ and control mice, consistent with the alterations detected in gonadal-intact female mice, it is possible that the contributing factors originated earlier during developmental stages, but after sexual maturation, and persisted after ovariectomy. The increased bone mass observed in POMC-C451A^f/f^ mice compared to controls, both in gonadal-intact and vehicle-treated ovariectomized mice, was similarly reported in untreated ovariectomized *Esr1*^Nkx2-1Cre^ mice (14). These mice lack total ERα signaling throughout the MBH, including the ARC neurons (14). Together with our findings, this highlights the critical role of estrogen signaling in ARC neurons in regulating female bone metabolism.

Loss of ERα signaling in POMC neurons has been shown to affect the negative feedback of sex steroids and the estrus cycle of female mice. In female POMC-C451A^f/f^ mice, elevated serum levels of E2, testosterone, and progesterone were observed, accompanied by a disrupted estrous cycle resembling that of POMC-ERαKO mice, characterized by shortened estrous and prolonged diestrus phase (21). Uterus weight normalized to body weight was unaffected in gonadal-intact POMC-C451A^f/f^ mice compared to controls, despite increased serum E2 concentrations, which may be explained by the counteracting effect of substantially elevated progesterone levels (51). Uterine responses to E2 reached a plateau at low-dose treatment, with no further effect between low- and high-dose treatments in either POMC-C451A^f/f^ or control mice. Our previous study demonstrated that membrane-initiated ERα signaling protects the uterus from supraphysiological E2 exposure (26). Findings from the current study indicate that mERα signaling in POMC neurons is not required for this protective effect.

The effect of lacking mERα signaling in POMC neurons was also evaluated in male mice. Sham-operated male POMC-C451A^f/f^ mice exhibited elevated serum testosterone concentrations compared to controls, suggesting that mERα signaling in POMC neurons contributes to the regulation of negative feedback of testosterone in males. In contrast to female mice, no significant skeletal differences were observed between sham-operated male POMC-C451A^f/f^ mice and controls, which is in line with findings from previous studies in male mice lacking ERα in hypothalamic neurons (14). Sex-dependent skeletal differences have also been reported in other ERα-related models, including those with ERα inactivation in osteoblast-lineage cells (52, 53), further supporting that ERα signaling exerts sexually dimorphic effects in bone regulation.

To address the gap in understanding the role of mERα signaling in POMC neurons in male mice under E2 stimulation, responses to low- and high-dose E2 were evaluated in orchiectomized male POMC-C451A^f/f^ mice and controls. The only significant difference observed between POMC-C451A^f/f^ mice and controls was increased femoral trabecular BMD in POMC-C451A^f/f^ mice and a trend toward an increase in BV/TV of L5 vertebra after high-dose E2 treatment. These findings suggest a possible alteration in trabecular sensitivity to supraphysiological E2 treatment in male mice in the absence of membrane-initiated ERα signaling in POMC neurons.

In conclusion, this study demonstrates that mERα signaling in POMC neurons regulates hormone homeostasis and bone metabolism, with more pronounced effects in female mice compared to male mice. Lacking mERα signaling in POMC neurons contributed to increased bone mass and enhanced trabecular response to E2 in the femur of females, while males showed minor changes. These findings highlight an important role of brain-derived mERα signaling in mediating effects on bone metabolism.

## Supplementary materials



## Declaration of interest

CO has two patents/patent applications in the field of probiotics and bone health. All other authors declare that they do not have any competing interests.

## Funding

This work was supported by the Swedish Research Council (2020-01840), the Swedish state under the agreement between the Swedish government and the county councils (ALF-agreement) (ALFGBG721581), the Gustaf V 80-years fund (FAI-2018-0466), the IngaBritt and Arne Lundberg Foundation (LU2017-0076), and the Novo Nordisk Foundation (26844).

## Author contribution statement

YJ, KH, CO, and MKL conducted the study design. YJ, KH, PH, KHN, JW, SMS, LL, and MKL were responsible for the acquisition of data, and YJ, KH, PH, LL, MKL, and CO performed the analysis and interpretation of data. YJ, KH, MKL, and CO wrote the main manuscript, and YJ, KH, and MKL prepared the figures. All authors reviewed the manuscript.

## Data availability

The data supporting the results of this study are available on request from the corresponding author. RNA-seq data, including raw data files (fastq format) and processed data files (matrix table of gene counts and differentially expressed genes in CSV format), are available in the Gene Expression Omnibus (GEO) repository, accession number GSE328990.
