# Supplementary material for: Membrane-initiated estrogen receptor-α signaling in the hypothalamus regulates trabecular bone in femur in female mice
Source: J Endocrinol. 2026 May 20;269(2):e250462. doi: 10.1530/JOE-25-0462 (PMC13192689; doi:10.1530/JOE-25-0462)
Supplement: Supplementary file 1 [file supplementary_materials.pdf]

Membrane-Initiated Estrogen Receptor-α Signaling in the Hypothalamus Regulates Trabecular Bone in Femur in Female Mice

Yiwen Jiang[1#], Karin Horkeby[1#], Petra Henning[1], Karin H Nilsson[1], Jianyao Wu[1], Lei Li[1], Sofia Movérare-Skrtic[1], Claes Ohlsson[1, 2], Marie K Lagerquist[1]

#These authors contributed equally to this study

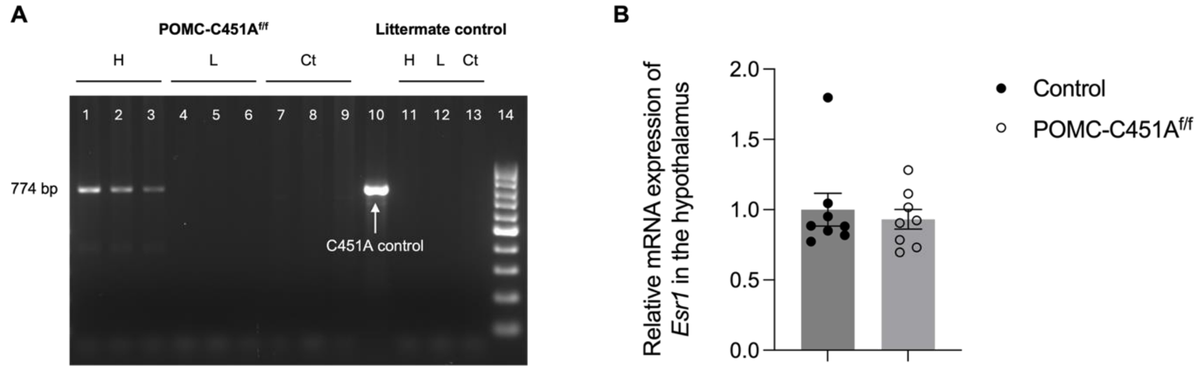

Supplementary figure 1. Validation of animal model. PCRs were performed on isolated DNA from hypothalamus (H), liver (L), and cortical bone (Ct) of POMC-C451A$^{f/f}$ and littermate control mice to assess the expression of mutated ERα-C451A (A). The relative mRNA expression pf estrogen receptor alpha (*Esr1*) was measured in the hypothalamus of POMC-C451A$^{f/f}$ and littermate controls (n=8) (B). Student's *t*-test was performed to verify the absence of significant differences between the two genotypes. Data are presented as mean ± SEM with individual values as dots.

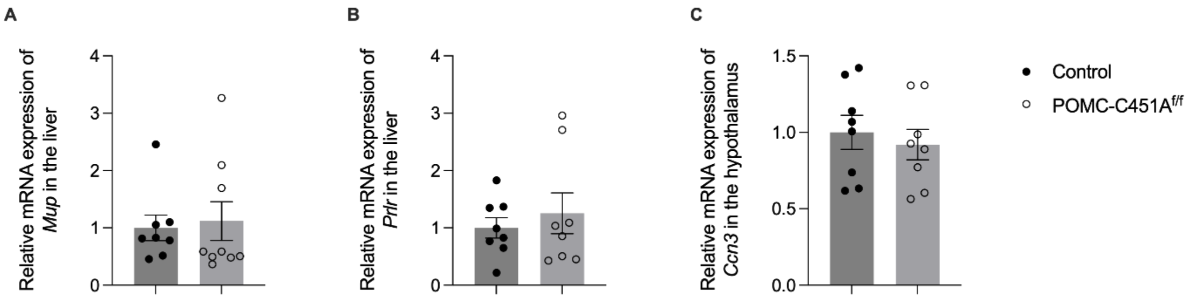

Supplementary figure 2. Bone-related gene expressions in the liver and the hypothalamus. Relative mRNA expressions of major urinary protein 3 (*Mup3*) (A) and prolactin receptor (*Prlr*) (B) were measured in the liver, and relative mRNA expression of nephroblastoma overexpressed gene (*Ccn3*) (C) was measured in the hypothalamus of gonadal-intact female POMC-C451A$^{f/f}$ and control (n=8-9). Student's *t*-test was performed to verify the absence of significant differences in *Mup* and *Ccn3* expressions between the two genotypes. Welch's *t*-test was used for comparing the expression of *Prlr* between genotypes. Data are presented as mean ± SEM with individual values as dots.

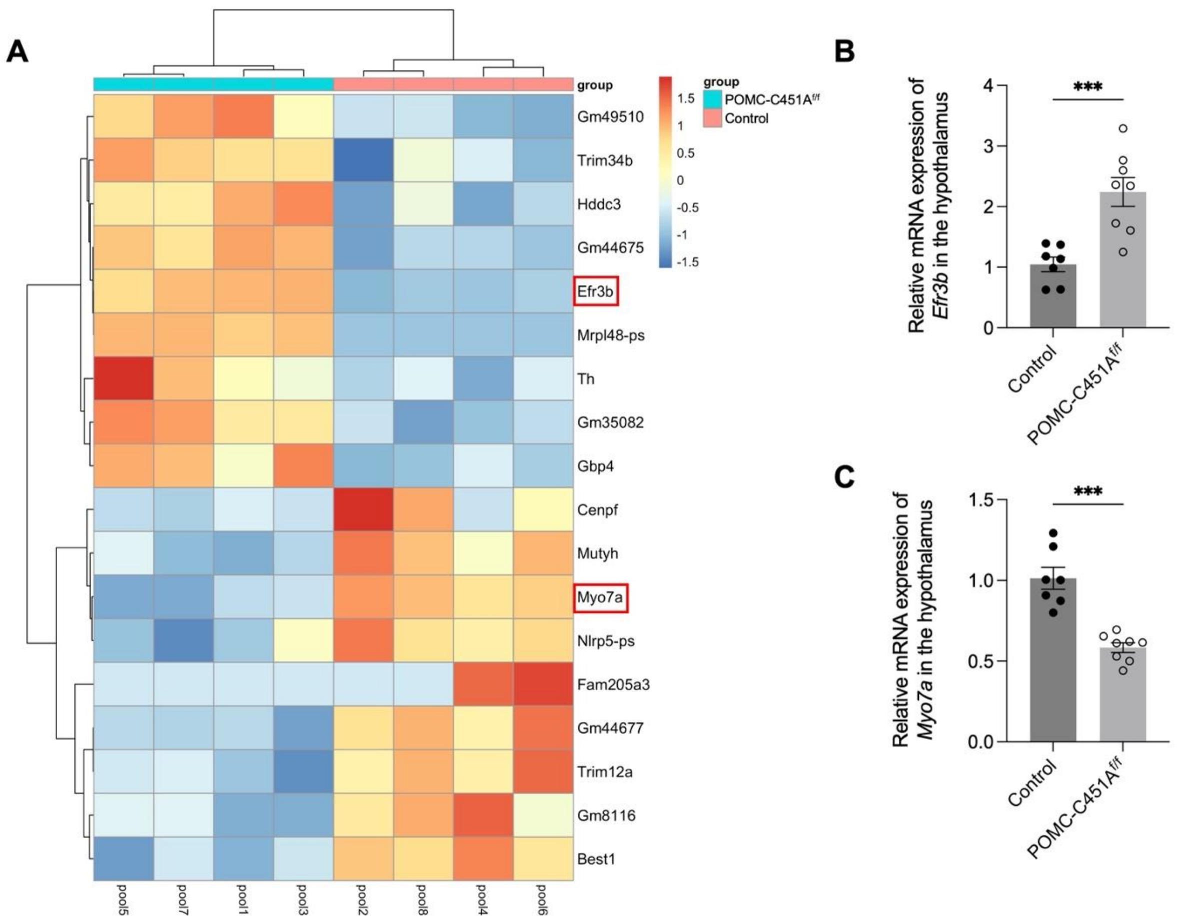

Supplementary figure 3. Differentially expressed genes in the hypothalamus. RNA sequencing of hypothalamic mRNA identified 18 differentially expressed genes (DEGs) between gonadal-intact female POMC-C451A$^{f/f}$ (n=4) and control mice (n=4), visualized in a heatmap and defined by a |log2(fold change)| > 0.5 and a false discovery rate (FDR) ≤ 0.05 (A). The relative mRNA expression of two bone-mass-related genes, Efr3 homolog b (*Efr3b*) (B) and myosin VII a (*Myo7a*) (C), was validated by qPCR in the hypothalamus of gonadal-intact female POMC-C451A$^{f/f}$ (n=8) and control (n=7). The differences between POMC-C451A$^{f/f}$ and control mice were analyzed by Student's *t* test. Data are presented as mean ± SEM with individual values as dots. ***p < 0.001.

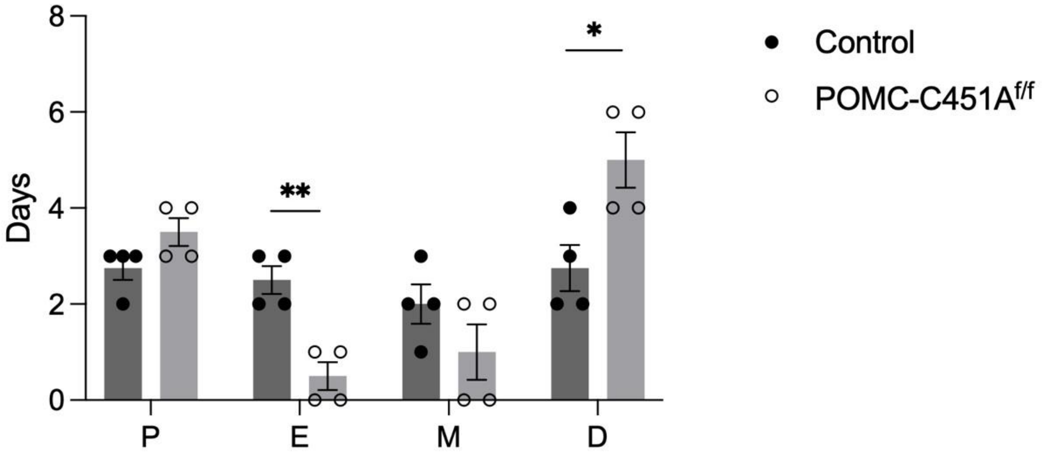

Supplementary figure 4. Disturbed estrous cycle in female POMC-C451A$^{f/f}$ mice. Estrous cycle was monitored via vaginal smear for ten consecutive days in female POMC-C451A$^{f/f}$ (n=4) and littermate control (n=4) mice. The differences between POMC-C451A$^{f/f}$ and control mice were analyzed by Student's *t* test. Data are presented as mean ± SEM with individual values as dots. *$p < 0.05$, **$p < 0.01$, proestrus; P, estrus; E, metestrus; M, diestrus; D.

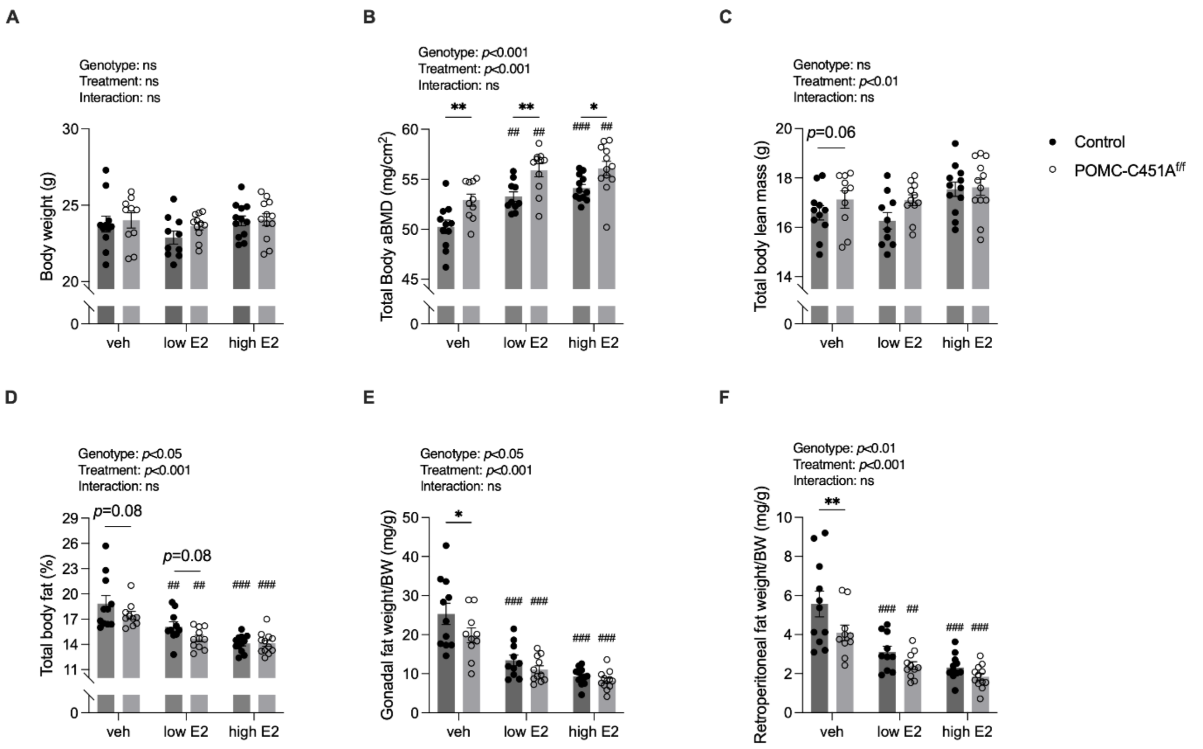

Supplementary figure 5. Female POMC-C451A$^{f/f}$ and control mice exhibited similar responses to estrogen in body composition and adipose tissue. Vehicle, low-dose estradiol (E2), and high-dose E2 were administrated subcutaneously in ovariectomized female POMC-C451A$^{f/f}$ and control. After three weeks of administration, all mice were weighed (A), and their body composition was assessed by dual-energy X-ray absorptiometry, including total body areal bone mineral density; aBMD (B), total body lean mass (C), and total body fat (D). At the time of termination, adipose tissue was dissected, weighed and normalized to their body weights, gonadal fat (E) and retroperitoneal fat (F). The differences between different treatments were analyzed by Two-way ANOVA test followed by Tukey's multiple comparisons test to compare differences between genotypes within each treatment group, and between vehicle- and E2-treated mice within the same genotype. A significant difference by Tukey's multiple comparisons test is indicated by $^{*}p < 0.05$, $^{**}p < 0.01$ (between genotypes within same treatment) and $^{\#\#}p < 0.01$, $^{\#\#\#}p < 0.001$ (between vehicle- and E2-treated mice within same genotype). Data are presented as mean ± SEM with individual values as dots. Vehicle control n=11, vehicle POMC-C451A$^{f/f}$ n=10, low-dose E2 control n=10, low-dose E2 POMC-C451A$^{f/f}$ n=11, high-dose E2 control n=12, high-dose E2 POMC-C451A$^{f/f}$ n=12.

Supplementary table 1. Phenotypes of female POMC-Cre mice and wildtype littermate controls.

	Wildtype	POMC-Cre
Body weight (g)	20.4 ± 2.3	20.6 ± 0.9
Uterus weight/BW (mg/g)	4.6 ± 2.2	4.9 ± 1.9
Gonadal fat weight/BW (mg/g)	12.7 ± 5.3	12.7 ± 3.5
Retroperitoneal fat weight/BW (mg/g)	2.2 ± 1.0	2.3 ± 1.0
Serum testosterone (pg/mL)	13.2 ± 7.8	13.6 ± 5.1
Serum estradiol (pg/mL)	1.0 ± 1.4	3.7 ± 5.5
Serum progesterone (pg/mL)	2053 ± 3223	1478 ± 2126
Total body aBMD (mg/cm^2)	48.0 ± 0.8	46.9 ± 1.5
Total body lean mass (g)	14.7 ± 1.7	14.9 ± 0.7
Total body fat (%)	15.6 ± 0.7	15.8 ± 0.8

Female POMC-Cre mice (n=7) and wildtype littermate controls (n=7) were sacrificed at eleven weeks of age. All mice were weighed and their uterus, gonadal fat tissue, and retroperitoneal fat tissue were collected, weighed, and normalized to their body weight (BW). Serum concentrations of testosterone, estradiol, and progesterone were measured by high-sensitivity liquid chromatography-tandem mass spectrometry. Body composition was assessed by dual-energy X-ray absorptiometry, including total body areal bone mineral density (aBMD), lean mass and fat. Student's *t*-test was performed to verify the absence of significant differences between female POMC-Cre mice and wildtype littermate controls. All values are presented as mean ± SD.

Supplementary table 2. Phenotypes of male POMC-Cre mice and wildtype littermate controls.

	Wildtype	POMC-Cre
Body weight (g)	27.0 ± 0.7	26.7 ± 1.2
Gonadal fat weight/BW (mg/g)	17.9 ± 7.0	15.1 ± 4.5
Retroperitoneal fat weight/BW (mg/g)	4.9 ± 2.7	3.9 ± 1.8
Serum testosterone (pg/mL)	408 ± 321	369 ± 173
Serum estradiol (pg/mL)	<LLOQ	<LLOQ
Serum progesterone (pg/mL)	705 ± 382	777 ± 588
Total body aBMD (mg/cm^2)	50.3 ± 1.7	50.8 ± 1.8
Total body lean mass (g)	20.7 ± 1.0	20.6 ± 1.1
Total body fat (%)	14.7 ± 3.5	13.6 ± 2.3

Male POMC-Cre mice (n=12) and wildtype littermate controls (n=8) were sacrificed at twelve weeks of age. All mice were weighed and their gonadal fat tissue, and retroperitoneal fat tissue were collected, weighed, and normalized to their body weight (BW). Serum concentrations of testosterone, estradiol, and progesterone were measured by high-sensitivity liquid chromatography-tandem mass spectrometry. The lower limit of quantification (LLOQ) for estradiol is 0.5 pg/mL. Body composition was assessed by dual-energy X-ray absorptiometry, including total body areal bone mineral density (aBMD), lean mass and fat. Student's *t*-test was performed to verify the absence of significant differences between male POMC-Cre mice and wildtype littermate controls. All values are presented as mean ± SD.

Supplementary table 3. Primers used for genotyping and validation.

Function	Direction	Sequencing (5′-3’)
**Genotyping**		
To detect the C451A-floxed allele	Forward	TTGAACCCTGACTTTCTCGG
	Reverse	TTCAAGGTGCTGGACAGAAAC
To detect the wildtype *Esr1* allele	Forward	CCACACAGTCCATATCTGCTAGA
	Reverse	TGTTGAATGTGGAGATCTGTGG
To detect the POMC-Cre allele	Forward	CCAGGCTAAGTGCCTTCTCTACA
	Reverse	AATGCTTCTGTCCGTTTGCCGGT
**Validation**		
To detect the mutated C451A allele	Forward	TTGAACCCTGACTTTCTCGG
	Reverse	ACCCGACATTAAATTCCCC
To measure *Efr3b* expression in the hypothalamus	Forward	ATGGGTACGTGTGTATTGCCA
	Reverse	GCTTTCCACGAAGAGGTTGAT
To measure *Myo7a* expression in the hypothalamus	Forward	CATCCGCCAGTACACCAACAA
	Reverse	TCCCCGCTGATAATACAGCAC
To measure expression of reference gene *Actb* in the hypothalamus	Forward	GGCTGTATTCCCCTCCATCG
	Reverse	CCAGTTGGTAACAATGCCATGT

Supplementary table 4. Differentially expressed genes in the hypothalamus of female gonadal-intact mice.

Gene	Gene type	Description	Log_2FC	*P* value	FDR
Gm49510	Protein coding	predicted gene 49510	1.34	2.53×10^{-5}	2.67×10^{-2}
Trim34b	Protein coding	tripartite motif-containing 34B	2.75	3.41×10^{-5}	3.27×10^{-2}
Hddc3	Protein coding	HD domain containing 3	0.82	1.47×10^{-9}	5.40×10^{-6}
Gm44675	TEC	predicted gene 44675	4.94	1.75×10^{-11}	7.72×10^{-8}
Efr3b	**Protein coding**	**EFR3 homolog B**	**1.02**	$\mathbf{1.61 \times 10^{-86}}$	$\mathbf{3.55 \times 10^{-82}}$
Mrpl48-ps	Processed pseudogene	mitochondrial ribosomal protein L48 pseudogene	9.11	2.42×10^{-18}	1.78×10^{-14}
Th	Protein coding	tyrosine hydroxylase	0.55	4.18×10^{-5}	3.79×10^{-2}
Gm35082	LncRNA	predicted gene 35082	1.51	1.45×10^{-7}	4.58×10^{-4}
Gbp4	Protein coding	guanylate binding protein 4	0.80	4.36×10^{-5}	3.79×10^{-2}
Cenpf	Protein coding	centromere protein F	-2.27	1.35×10^{-5}	1.76×10^{-2}
Mutyh	Protein coding	mutY DNA glycosylase	-0.78	2.51×10^{-5}	2.66×10^{-2}
Myo7a	**Protein coding**	**myosin VIIA**	**-0.77**	$\mathbf{4.64 \times 10^{-20}}$	$\mathbf{5.12 \times 10^{-16}}$
Nlrp5-ps	Processed pseudogene	NLR family, pyrin domain containing 5 pseudogene	-1.11	5×10^{-5}	3.81×10^{-2}
Fam205a3	Protein coding	spermatogenesis associated 31 subfamily F member 1C	-22.8	1.73×10^{-11}	7.72×10^{-8}
Gm44677	TEC	predicted gene 44677	-1.33	1.22×10^{-6}	2.69×10^{-3}
Trim12a	Protein coding	tripartite motif-containing 12A	-1.00	2.29×10^{-5}	2.66×10^{-2}
Gm8116	Processed pseudogene	predicted gene 8116	-3.96	6.93×10^{-6}	1.09×10^{-2}
Best1	Protein coding	bestrophin 1	-0.64	1.03×10^{-5}	1.41×10^{-2}

The 18 differentially expressed genes (DEGs) identified by RNA-sequencing in hypothylamus of female gonadal-intact POMC-C451A$^{f/f}$ compared with controls that met the criteria of FDR ≤ 0.05 and |log2FC| > 0.5. False discovery rate; FDR, to be experimentally confirmed; TEC.
